# Author Correction: Molecular basis for the inhibition of the methyl-lysine binding function of 53BP1 by TIRR

**DOI:** 10.1038/s41467-018-08086-w

**Published:** 2019-01-08

**Authors:** Jiaxu Wang, Zenglin Yuan, Yaqi Cui, Rong Xie, Guang Yang, Muzaffer A. Kassab, Mengxi Wang, Yinliang Ma, Chen Wu, Xiaochun Yu, Xiuhua Liu

**Affiliations:** 1 College of Life Sciences, Hebei University, Baoding, 071000 Hebei China; 2 State Key Laboratory of Microbial Technology, Shandong University, Jinan, 250100 Shandong China; 3 Department of Cancer Genetics and Epigenetics, Beckman Research Institute, City of Hope, 1500 East Duarte Road, 91010 Duarte, CA USA

Correction to: *Nature Communications*; 10.1038/s41467-018-05174-9; published online 12 July 2018

The original version of this Article contained an error in the author affiliations.

Xiaochun Yu was incorrectly associated with College of Life Sciences, Hebei University, Baoding 071000 Hebei, China.

This has now been corrected in both the PDF and HTML versions of the Article.

