# Supplementary material for: Molecular basis for the inhibition of the methyl-lysine binding function of 53BP1 by TIRR
Source: Nat Commun. 2018 Jul 12;9:2689. doi: 10.1038/s41467-018-05174-9 (PMC6043480; doi:10.1038/s41467-018-05174-9)
Supplement: Supplementary file 1 — Supplementary Information [file 41467_2018_5174_MOESM1_ESM.pdf]

Molecular basis for the inhibition of the methyl-lysine binding function of 53BP1 by TIRR

Wang et al

Supplementary Information

Figure S1

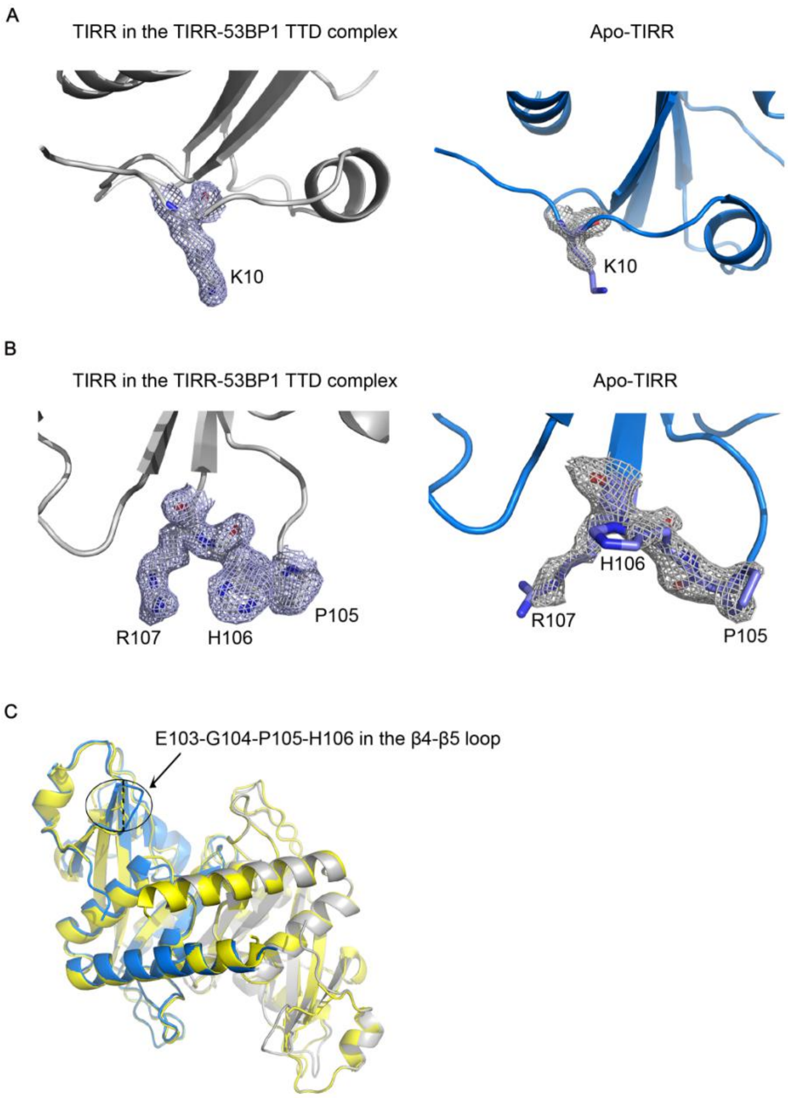

Supplementary Fig. 1. Structural analysis of TIRR. (A) The 2*Fo-Fc* electron density map overlaying Lys^{10} from TIRR (contoured at 1.0 σ level). (B) The 2*Fo-Fc* electron density maps covering the three residues Pro^{105}-His^{106}-Arg^{107} and Lys^{10} from TIRR (contoured at 1.0 σ level). (C) Structure comparison of TIRR monomer in the TIRR-53BP1 complex and apo-TIRR (PDB ID: 3KVH). The TIRR monomer in the TIRR-53BP1 complex is in gray and blue. Apo-TIRR is in yellow. The overall folding of TIRR monomer is similar to apo-TIRR, except four residues (Glu^{103}-Gly^{104}-Pro^{105}-His^{106}) from β4-β5 loop in the apo-TIRR form are in disordered conformation, which is marked by a black dashed line.

Figure S2

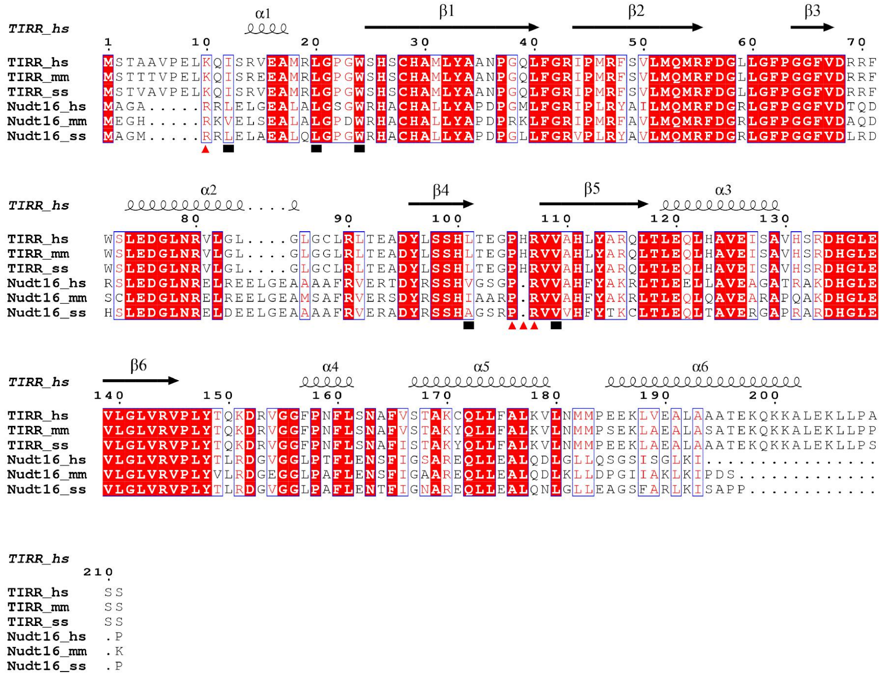

Supplementary Fig. 2. Sequence alignment of TIRR_hs, TIRR_mm, TIRR_ss, Nudt16_hs, Nudt16_mm and Nudt16_ss was performed using T-Coffee and the results were shown via ESPript. The residues involved in the TTD binding are underlined and in red triangles. TIRR_hs, TIRR_mm, TIRR_ss are TIRRs from *Homo sapiens*, *Mus musculus* and *Sus scrofa* respectively. Nudt16_hs, Nudt16_mm and Nudt16_ss are Nudt16 from *Homo sapiens*, *Mus musculus* and *Sus scrofa* respectively.

Figure S3

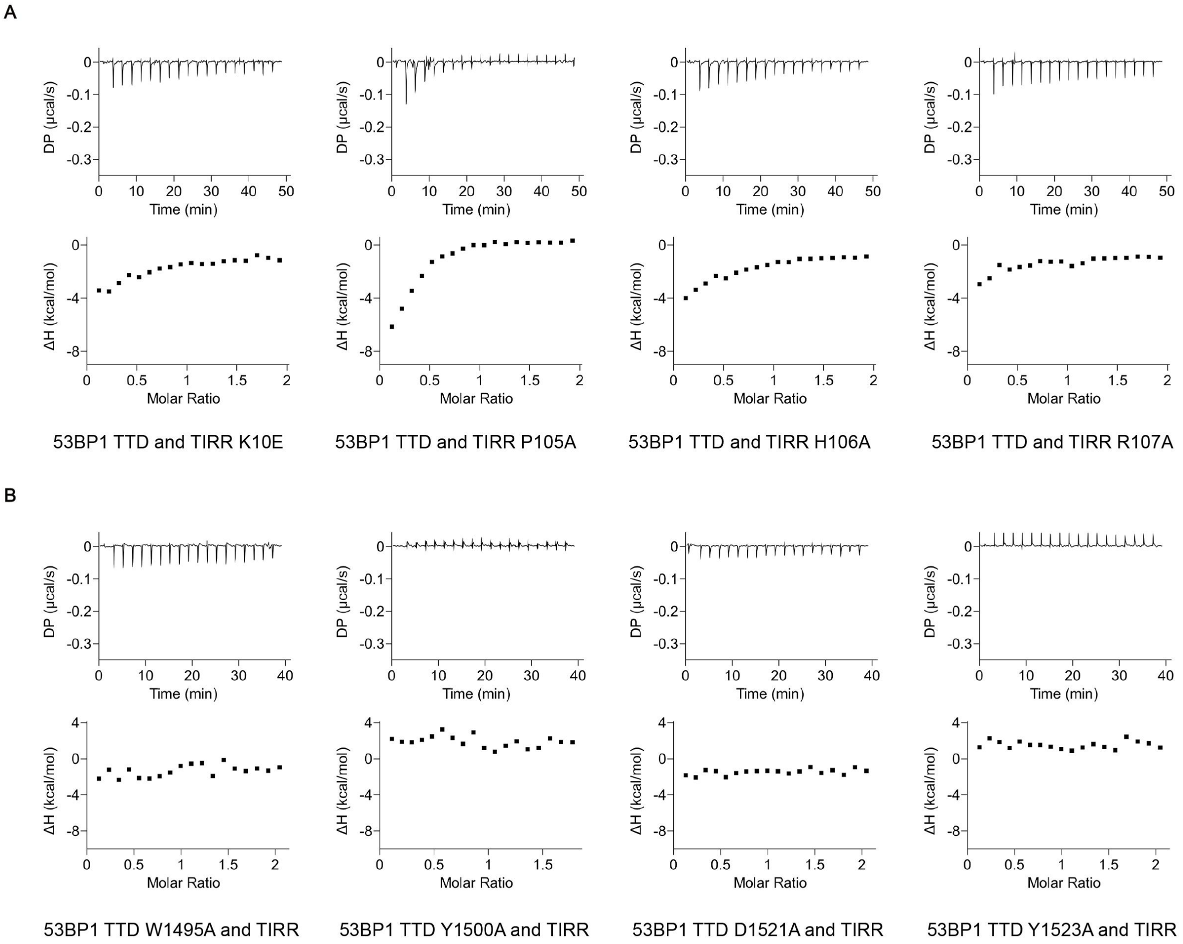

Supplementary Fig. 3. Binding affinity between 53BP1 TTD and TIRR**.** (A) The binding affinities between 53BP1 TTD and TIRR K10E, P105A, H106A and R107A (from left to right) were examined by ITC. (B) The binding affinities between TIRR and 53BP1 TTD W1495A, Y1500A, D1521A and Y1523A (from left to right) were examined by ITC.

Figure S4

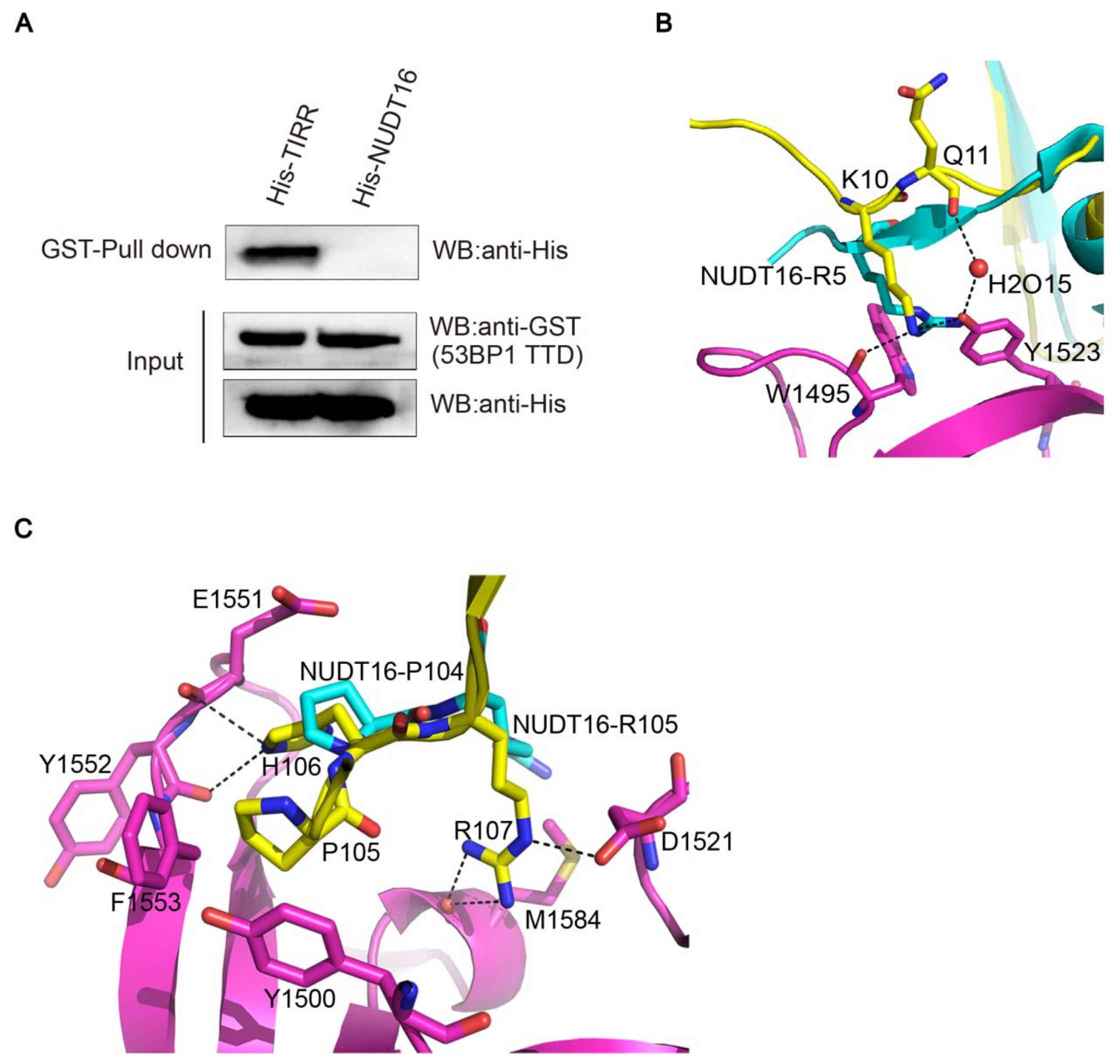

Supplementary Fig. 4. NUDT16 does not directly bind to 53BP1 TTD. (A) TIRR but not NUDT16 direct binds to 53BP1 TTD. In vitro interaction between Nudt16 and 53BP1 TTD was examined by GST pull down assay. (B and C) Superposition of the TIRR (yellow) in TIRR-TTD complex with NUDT16 (blue) in the N-terminus loop (B) and β4-β5 loop (C). The key residues Lys^{10}, Pro^{105}, His^{106} and Arg^{107} from TIRR are in yellow stick. The corresponding residues Arg^{5}, Pro^{104} and Arg^{106} from NUDT16 are in blue stick.

Figure S5

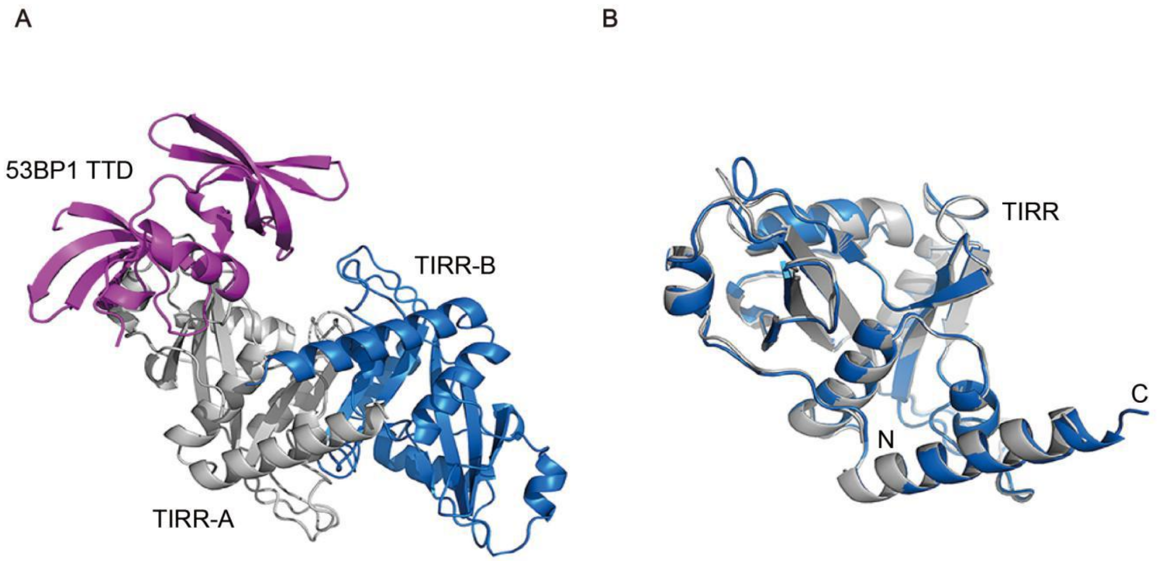

Supplementary Fig. 5. TIRR forms homodimer in the complex with 53BP1 TTD. (A) TIRR-A and TIRR-B are displayed as gray and blue respectively, TTD is shown as magenta. (B) Superposition of TIRR-A with TIRR-B.

Figure S6

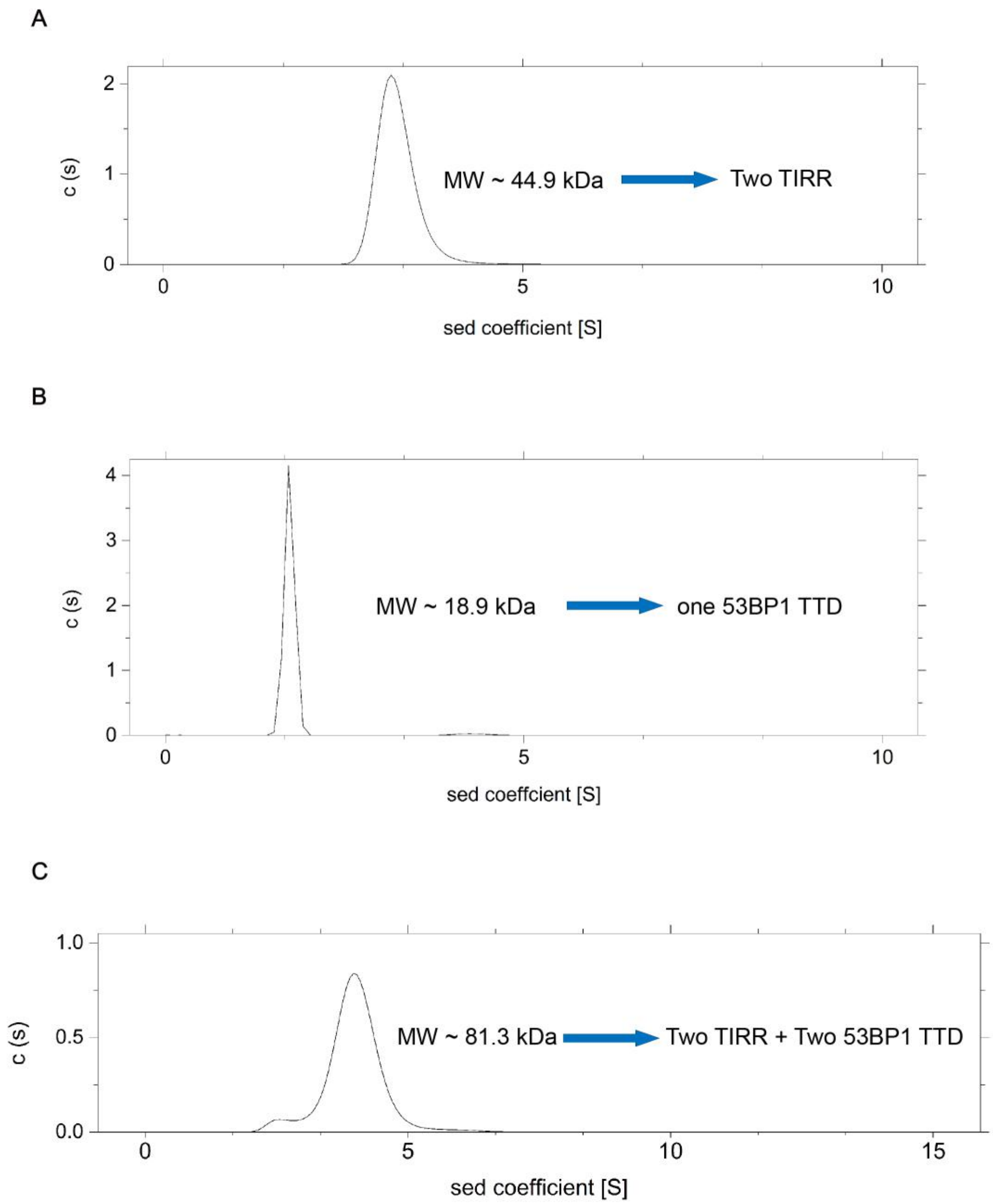

Supplementary Fig. 6. Analytical ultracentrifugation analysis. (A) The molecular weight of TIRR was determined to be 44.5 kDa, indicating that TIRR (22.9 kDa) exists as a homodimer in solution. (B) The molecular weight of 53BP1 TTD was measured to be 19.3 kDa, indicating that 53BP1 TTD (19.3 kDa) exists as monomer in solution. (C) Molecular weight of TIRR-53BP1 TTD complex was determined to be 81.3 kDa, indicating that this protein complex contains two TIRR molecules and two 53BP1 TTD molecules.

Figure S7

A

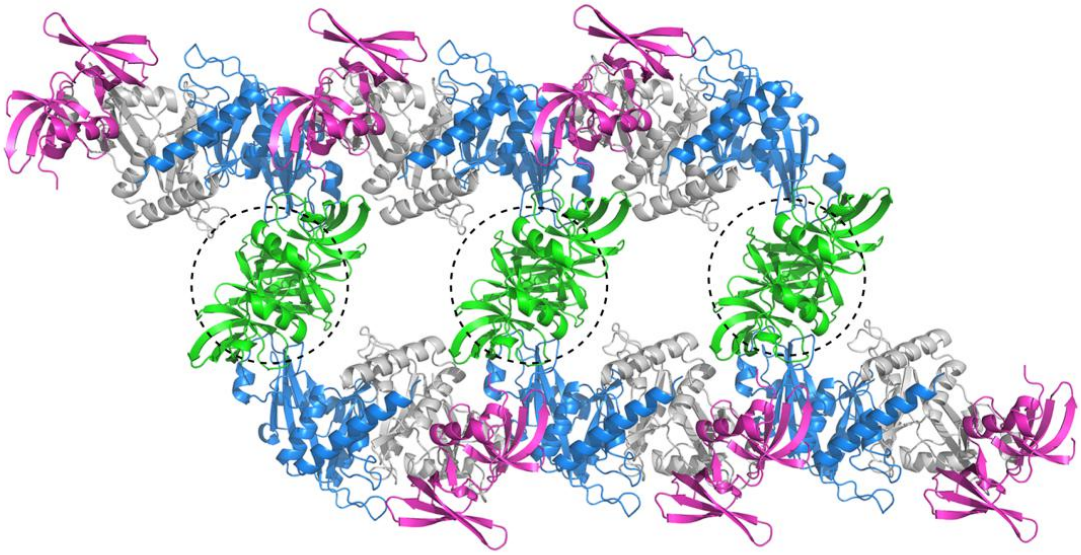

B

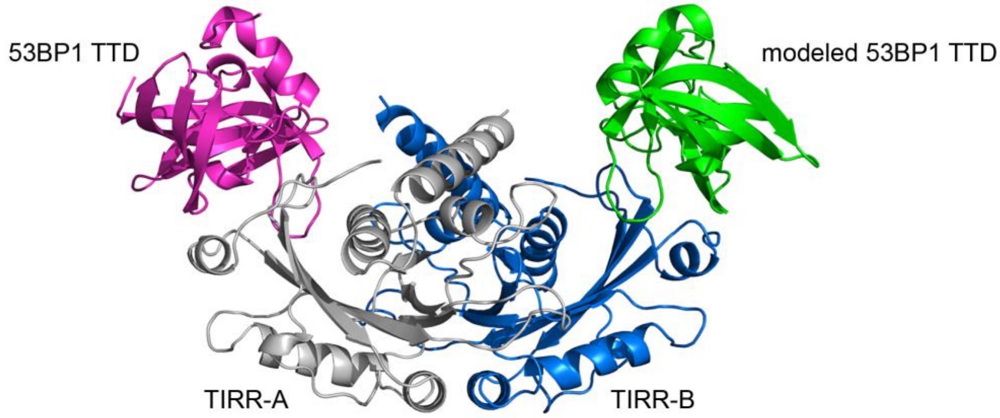

Supplementary Fig. 7. Modeling the second TTD molecule into the crystal structure of TIRR-TTD complex. (A) The modeled TTD molecules clash seriously in the crystal lattice. The clashed regions are marked using black dashed lines. (B) TIRR-53BP1 complex consists of two TIRR molecules and two TTD molecules. The TTD molecule (magenta) binds to the TIRR monomer A (gray), another TTD molecule (green) was modeled and binds to TIRR monomer B (blue).

Figure S8

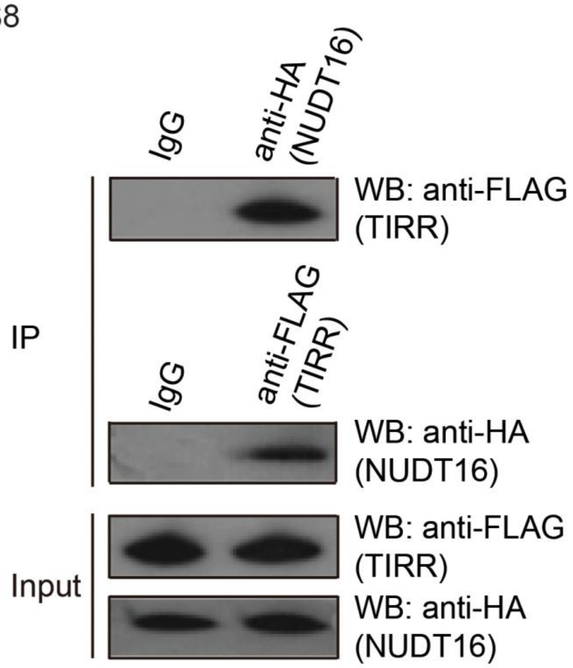

Supplementary Fig. 8. TIRR is associated with NUDT16. 293T cells were expressed SFB-TIRR and HA-NUDT16. The interaction between TIRR and NUDT16 was examined by IP and Western blot with indicated antibodies.

Figure S9

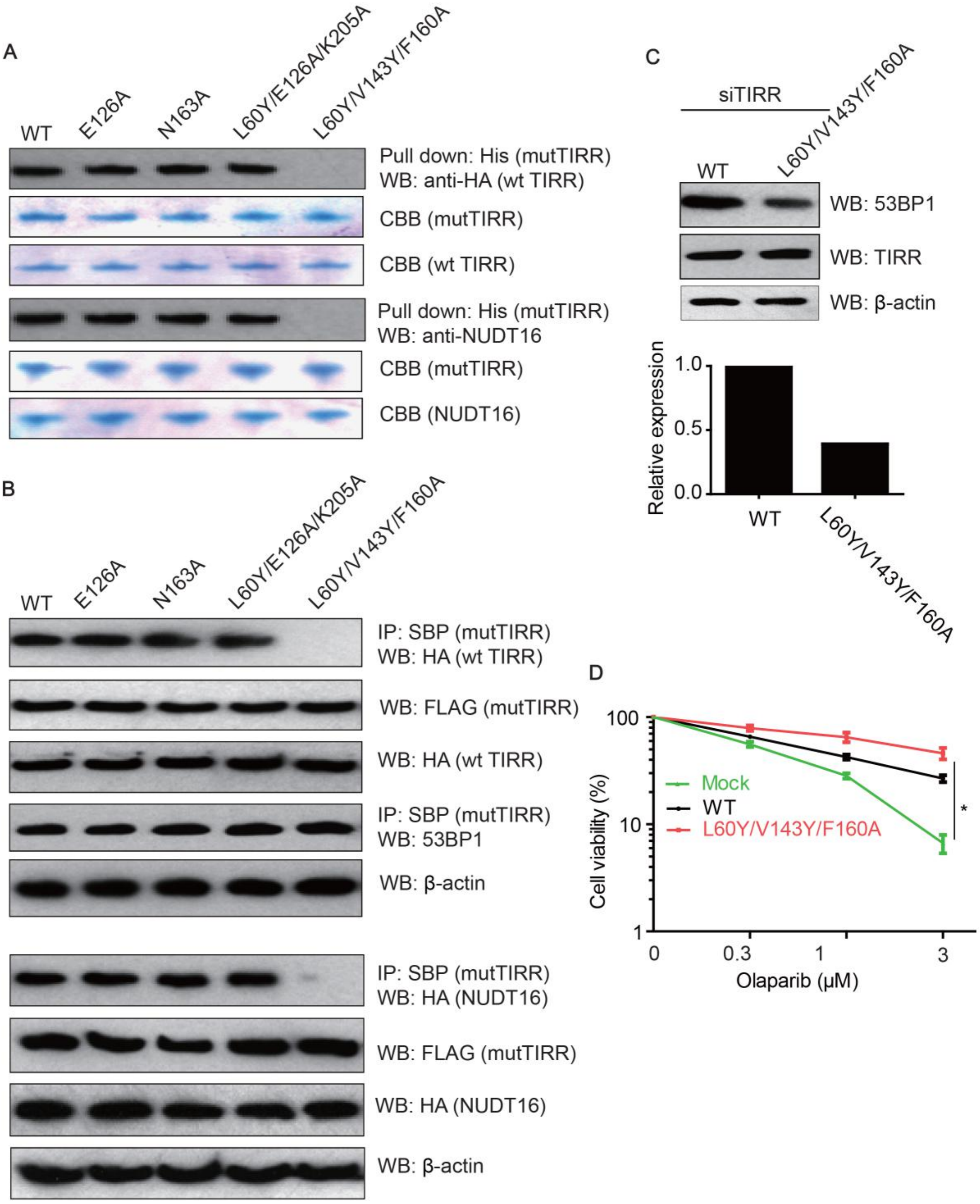
A
WT
E126A
N163A
L60Y/E126A/K205A
L60Y/V143Y/F160A
Pull down: His (mutTIRR)
WB: anti-HA (wt TIRR)
CBB (mutTIRR)
CBB (wt TIRR)
Pull down: His (mutTIRR)
WB: anti-NUDT16
CBB (mutTIRR)
CBB (NUDT16)
B
WT
E126A
N163A
L60Y/E126A/K205A
L60Y/V143Y/F160A
IP: SBP (mutTIRR)
WB: HA (wt TIRR)
WB: FLAG (mutTIRR)
WB: HA (wt TIRR)
IP: SBP (mutTIRR)
WB: 53BP1
WB: β-actin
IP: SBP (mutTIRR)
WB: HA (NUDT16)
WB: FLAG (mutTIRR)
WB: HA (NUDT16)
WB: β-actin
C
siTIRR
WT
L60Y/V143Y/F160A
WB: 53BP1
WB: TIRR
WB: β-actin
Relative expression
1.0
0.5
0.0
WT
L60Y/V143Y/F160A
D
Cell viability (%)
100
10
1
Mock
WT
L60Y/V143Y/F160A
*
0
0.3
1
3
Olaparib (μM)

Figure S9

E

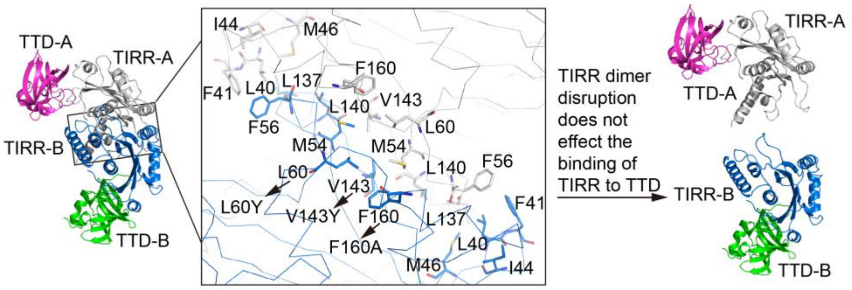

F

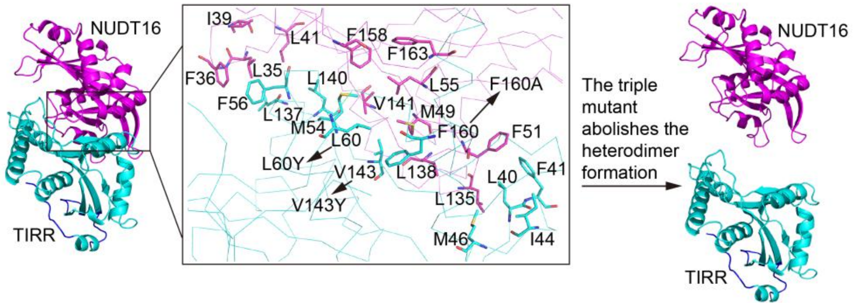

Supplementary Fig. 9. Dimer formation of TIRR. (A) The L60Y/V143Y/F160A mutation abolishes the TIRR homodimer or TIRR/NUDT16 heterodimer. The recombinant HIS tagged TIRR mutants were incubated HA tagged wild type TIRR or untagged NUDT16. The pull down and Western blot assays were performed. (B) SBP-TIRR mutants were co-expressed with HA-TIRR or HA-NUDT16 in U2OS cells. The co-IP and Western blot assays were performed with indicated antibodies. (C) U2OS cells were treated with siTIRR to knockdown endogenous TIRR. Wild type TIRR or the L60Y/V143Y/F160A mutant was reintroduced into the cells. The expression of 53BP1 and ectopic TIRR were examined by Western blot. β-actin was used as the protein loading control. (D) Wild type TIRR or the L60Y/V143Y/F160A mutant was expressed in UWB1 (BRCA1-deficient) cells. The cells were treated with indicated dose of olaparib (PARP inhibitor) for 6 days. Cell viability was examined. Data are represented as the mean ± s.d. (n=3). *: $p < 0.05$. (E) Structure of the TIRR-TTD complex dimer interface. The zoomed region shows the hydrophobic residues at the TIRR dimer interface. (F) The TIRR-NUDT16 heterodimer model and the heterodimer interface. The zoomed region shows the hydrophobic residues at the interface of the heterodimer.

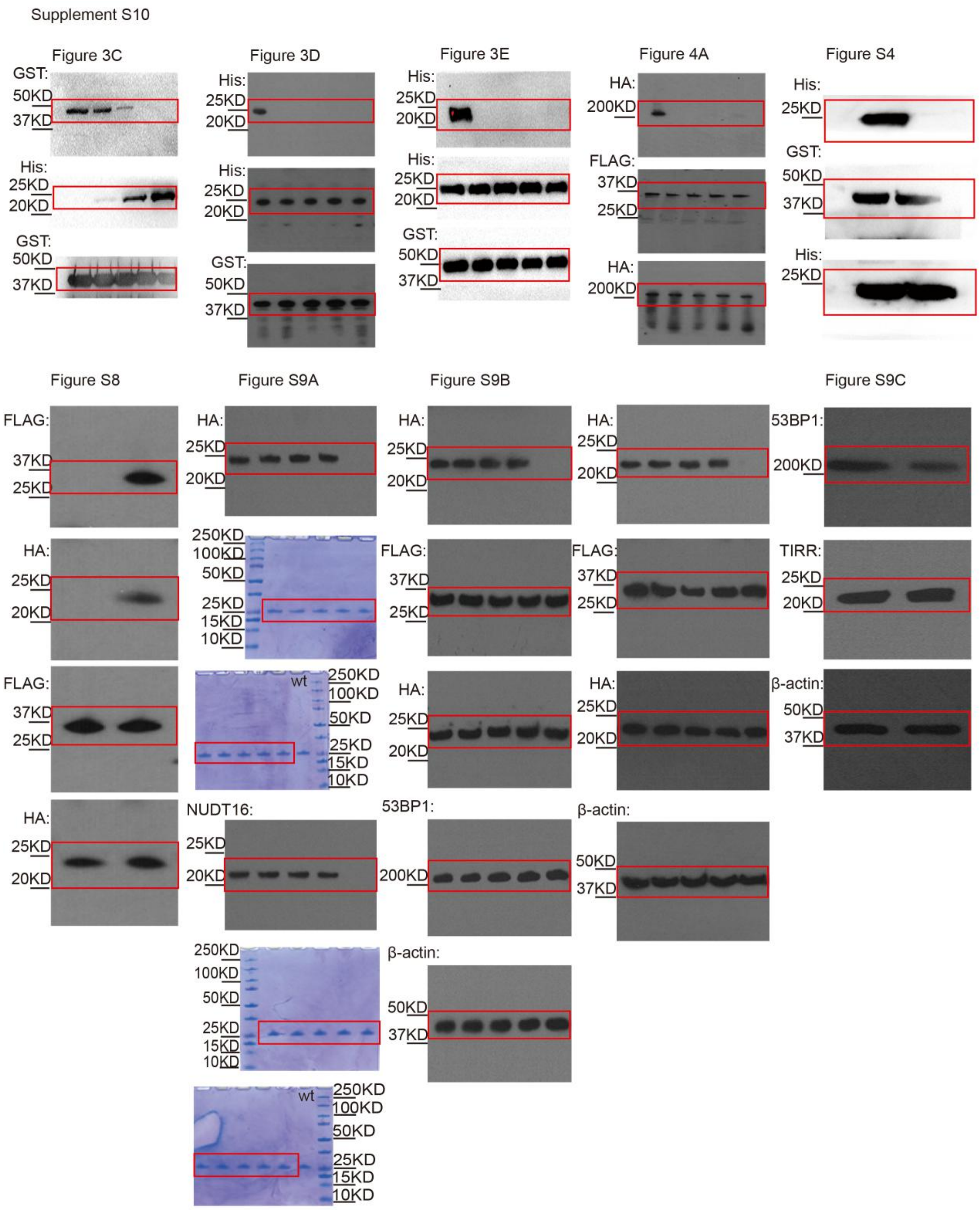

Supplementary Fig. 10. Original images of western blots and gels shown in this study.

Supplementary Table 1. List of primers used in TIRR construct

Primer name	Sequence (5’ - 3’)
Human TIRR 1 F	CGGGATCCATGTCGACGGCGGCGGTT
Human TIRR 6 F	CGGGATCCGTTCCGGAGCTGAAGCAGATC
Human TIRR 211 R	ACGTCACTCGAGTCAAGAGGAGGCCGGGAG
Human TIRR K10E F	GTTCCGGAGCTGGAGCAGATCAGCCGG
Human TIRR K10E R	CCGGCTGATCTGCTCCAGCTCCGGAAC
Human TIRR P105A F	CTGACCGAGGGCGCACACCGCGTCGTG
Human TIRR P105A R	CACGACGCGGTGTGCGCCCTCGGTCAG
Human TIRR H106A F	ACCGAGGGCCCAGCCCGCGTCGTGGCG
Human TIRR H106A R	CGCCACGACGCGGGCTGGGCCCTCGGT
Human TIRR R107A F	AGGGCCCACACGCCGTCGTGGCGCA
Human TIRR R107A R	TGCGCCACGACGGCGTGTGGGCCCT
Human TIRR L60Y R	TTCGACGGGCTGTATGGCTTCCCCGGG
Human TIRR L60Y F	CCCGGGGAAGCCATACAGCCCGTCGAA
Human TIRR V143Y R	GTGCTGGGCCTCTATCGGGTCCCGCTG
Human TIRR V143Y F	CAGCGGGACCCGATAGAGGCCCAGCAC
Human TIRR F160A F	GGCTTCCCCAACGCCCTGAGCAACGCC
Human TIRR F160A R	GGCGTTGCTCAGGGCGTTGGGGAAGCC
Human TIRR E126A F	CTGCACGCCGTGGCGATCAGCGCGGTG
Human TIRR E126A R	CACCGCGCTGATCGCCACGGCGTGCAG
Human TIRR K205A F	AAGGCCCTGGAGGCGTTGCTCCCGGCC
Human TIRR K205A R	GGCCGGGAGCAACGCCTCCAGGGCCTT
Human TIRR N163A F	AACTTCCTGAGCGCCGCCTTCGTGAGC
Human TIRR N163A R	GCTCACGAAGGCGGCGCTCAGGAAGTT

Supplementary Table 2. List of primers used in 53BP1 construct

Primer name	Sequence (5' - 3')
Human 53BP1 1459 F	CGGGATCCCGTAGTGACTCTCCAGAAATTC
Human 53BP1 1643 R	ACGTCACTCGAGCTAGCTGACGTTACTGCGCCGTTT
Human 53BP1 W1495A F	GTTGTAGCCAAGGCGTCATCCAATGGC
Human 53BP1 W1495A R	GCCATTGGATGACGCCTTGGCTACAAC
Human 53BP1 D1500A F	TCATCCAATGGCGCCTTTTACTCTGGG
Human 53BP1 D1500A R	CCCAGAGTAAAAGGCGCCATTGGATGA
Human 53BP1 W1521A F	ATTGCTCTTTGATGCTGGGTACGAATGTG
Human 53BP1 W1521A R	CACATTCGTACCCAGCATCAAAGAGCAAT
Human 53BP1 W1523A F	CTTTGATGATGGGGCCGAATGTGATGTG
Human 53BP1 W1523A R	CACATCACATTCGGCCCCATCATCAAAG
Human 53BP1 W1553A F	GAGGATGAGTATGCCAGTGCAGGAGTG
Human 53BP1 W1553A R	CACTCCTGCACTGGCATACTCATCCTC

Supplementary Table 3. List of antibodies

Antibody	Company	(Cat.No.)	Source	Application	Dilution
anti-GST	ZSGB-BIO	TA-02	Mouse monoclonal	Immunoblot	1:1000
anti-His	ZSGB-BIO	TA-03	Mouse monoclonal	Immunoblot	1:1000
anti-HA	BioLegend	MMS-101P	Mouse monoclonal	Immunoblot	1:1000
anti-Flag	Sigma	F3165	Mouse monoclonal	Immunoblot	1:1000
anti-53BP1	Cell Signaling Technology	4937s	Rabbit polyclonal	Immunoblot Immunofluorescence	1:1000
anti-RIF1	GeneTex	GTX131889	Rabbit polyclonal	Immunofluorescence	1: 500
anti-γH2AX	Milipore	16-202A	Mouse monoclonal	Immunofluorescence	1:1000
anti-PRA2	Cell Signaling Technology	2208S	Rabbit polyclonal	Immunofluorescence	1:100
anti-RAD51	Novus Biologicals	NBP2-32622	Rabbit polyclonal	Immunofluorescence	1: 200
anti-NUDT16	proteintech	12889-1-AP	Rabbit polyclonal	Immunoblot	1:1000
anti-β-actin	Sigma	A5441	Mouse monoclonal	Immunoblot	1:2000
